# A Study of the Compressive Behavior of Recycled Rubber Concrete Reinforced with Hybrid Fibers

**DOI:** 10.3390/ma16134731

**Published:** 2023-06-30

**Authors:** Xiaohui Li, Lijuan Li, Yingming Zheng, Yanlong Li, Zijiang Chen, Jie Xiao, Min Yuan, Jian Zhang, Zezhou Pan, Zhe Xiong

**Affiliations:** 1 School of Civil and Transportation Engineering, Guangdong University of Technology, Guangzhou 510006, China; 2 Guangzhou Building Development and Construction Co., Ltd., Guangzhou 510000, China; 3 China Railway Guangzhou Engineering Group Co., Ltd., Guangzhou 511459, China

**Keywords:** compressive behavior, glass fibers, hybrid fibers, recycled rubber concrete, steel fibers

## Abstract

With the development of the automotive industry, a large amount of waste rubber is produced every year. The application and development of recycled rubber concrete (RRC) can effectively reduce ‘black pollution’ caused by waste rubber. However, the addition of recycled rubber particles can lead to a decrease in the compressive behavior of concrete. Previous research has demonstrated that by preventing crack growth, fiber addition can increase the strength and ductility of concrete. In this work, a total of 28 RRC mixes are designed, and the compressive behavior of RRC reinforced by steel fibers (SFs) and glass fibers (GFs) is investigated. The workability of fresh RRC can be negatively impacted by an increase in both fiber contents, with the GF content having a more notable effect. With the addition of fibers, the maximum increase rates for the compressive strength, elastic modulus, strain at peak stress, and compressive toughness were 27%, 8%, 45%, and 152%, respectively. A constitutive model is concurrently put forward to forecast the stress–strain curves of RRC with various fiber contents. These findings indicate that the maximum improvement in compressive behavior is achieved when the GF content was 0.4% and the SF content was 1.2%. The proposed constitutive model can be used to predict the stress–strain curve of hybrid fiber-reinforced recycled rubber concrete (HFRRRC).

## 1. Introduction

Globally, concrete is still the most widely utilized building material due to its excellent plasticity, safety, and durability. The volume proportions of coarse and aggregates in concrete are approximately 70%. Concrete is widely utilized, which has led to an overuse of natural aggregates, such as river sand, which has harmed the biological environment. Therefore, in recent years, many researchers have investigated materials suitable for replacing natural aggregates with concrete [1,2]. The most commonly used methods involve crushed waste tires [3], crushed abandoned concrete [4], and sea sand [5].

Tire trash is produced in significant quantities each year as a result of the automobile industry’s fast expansion. Although different recycling procedures consume some of the waste tires, a great deal of used tire garbage is still landfilled every year. However, owing to increasing costs, the landfill process for solid waste has become unacceptable. Therefore, to minimize the impact of waste tires on the environment, research has been conducted to assess the physical characteristics and toughness of concrete made with recycled tire rubber in place of conventional aggregates [6]. Recycled rubber (RR) can be added to ordinary concrete to enhance certain qualities including energy absorption, freeze–thaw resistance, and hardness [7,8,9,10]. However, plain concrete’s strength can be greatly decreased by a large rubber component, and optimal rubber content should be considered when producing recycled rubber concrete (RRC) [11,12,13]. Additionally, using elastic rubber might result in plain concrete drying out with more shrinkage since soft rubber is more likely to flex under internal shrinkage stress than conventional coarse and fine particles [14,15]. These negative effects limit the widespread application of RRC despite its advantages and environmental benefits.

Strength and stiffness are two important indicators that need to be considered in structural design [16]. To enhance the properties of RRC, reinforcement of RRC through chemical or physical reinforcement can be performed. Rubber can have its surface chemically treated to enhance the mechanical behaviors of RRC. The most widely used method involves immersing rubber particles in a NaOH solution before use in concrete mixtures. Through this process, the rubber loses its hydrophobicity and becomes hydrophilic. The result is the creation of denser cement hydration products surrounding the rubber particles, which helps the rubber and cement matrix to better bond with each other [17,18,19]. The addition of fibers that do not chemically react with RRC to rubber concrete can provide physical reinforcement. Different types of fibers, such as steel, glass, polyester, polypropylene, nylon, rayon, carbon, basalt, cotton, and sisal, have been used to reinforce concrete [20,21]. Currently, fiber-reinforced concrete is normally produced using just one type of fiber, and its effectiveness is rather moderate [22,23]. The insertion of single fibers does not appreciably improve the mechanical characteristics of concrete since cracking and failure of concrete occur on several scales [24]. Some researchers have proven that the hybridization of two or more types of fibers can improve the ultimate strength, strain capacity, and strain-hardening behavior of concrete. Different hybridization methods include combining different fiber lengths, diameters, elastic moduli, and tensile strengths [25,26,27]. Hybridization methods can be classified into two types. (1) Hybridization based on the size of the fiber (length and diameter) due to the different fiber sizes and small-sized fibers bridge microcracks to control their aggregation, whereas larger-sized fibers prevent the expansion of macroscopic cracks. Concrete strength and fracture toughness may both be improved by managing macro- and microcracks, respectively. Owing to this synergistic mechanism, the enhancement in ductility mainly depends on long fibers. The specific surface area, which may be characterized as the surface area per unit mass, is commonly used to describe the fiber’s size. (2) Hybridization based on the elastic modulus of the fiber. Because two types of fibers with different flexibilities are added to concrete, stronger and harder fibers enhance the stress and ultimate stress when cracks appear, whereas relatively softer fibers improve the toughness and strain capacity of cracked concrete. In recent studies, concrete reinforced with hybrid fibers has been used to achieve higher strength, deformation capacity, and durability than concrete reinforced by a single fiber [28,29]. The two most prevalent types of fibers used in fiber-reinforced concrete are glass fibers (GFs) and steel fibers (SFs). However, there is relatively little research on hybrid SF- and GF-reinforced concrete, especially for concrete that uses recycled aggregates, such as recycled rubber.

The hybridization of harder and softer fibers in concrete has a good synergistic effect, which improves the strength and ductility of concrete. The size, shape, type, and volume content of the fiber are all connected to this enhancement. Although adding SFs and GFs at the same time to increase the concrete’s strength and ductility appears feasible, there has not been many studies on the mechanical characteristics of RRC incorporating SFs and GFs. At the same time, a constitutive model for two types of fibers was proposed. Therefore, this study investigated the axial compression behavior of concrete containing SFs and GFs. The research results were used to analyze the comprehensive impact of SFs and GFs on the compressive behavior of RRC. The findings of this study offer crucial recommendations for the creation and use of RRC.

## 2. Materials and Methods

This study tested the workability and compressive behavior of hybrid fiber-reinforced recycled rubber concrete (HFRRRC) through slump and axial compressive tests.

### 2.1. Materials

**Figure 1:**
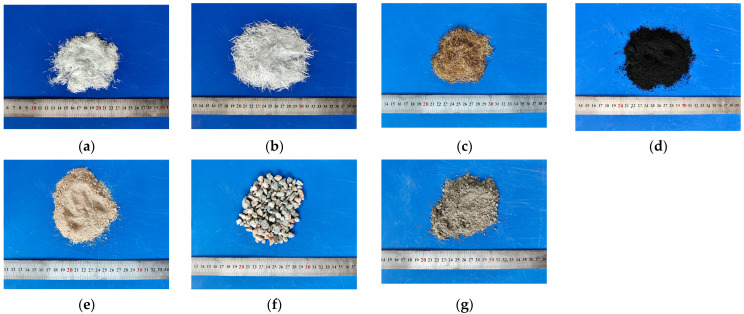
Raw materials of HFRRRC: (**a**) 6 mm GF; (**b**) 12 mm GF; (**c**) SF; (**d**) sand; (**e**) RR; (**f**) CA; (**g**) cement.

**Figure 2:**
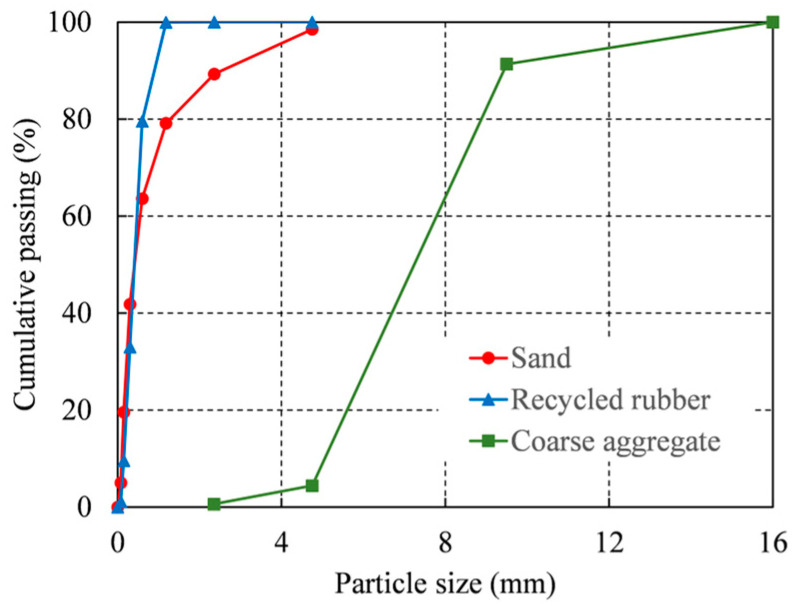
Particle size distribution of aggregates.

Cement, water, RR, sand, coarse aggregate, SFs, and GFs are combined to produce HFRRRC, as shown in Figure 1. Ordinary Portland cement with a strength grade of 42.5 MPa and a specific gravity of 3.11 was used. Tap water with a specific gravity of 1.00 was obtained from the laboratory. River sand with a maximum particle size of 5 mm and 20 mesh recycled rubber were used as fine aggregates (FAs) in this study. According to the standard GB/T 14684-2011 [30], the properties of the sand were obtained from the tests, as listed in Table 1. The properties of the recycled rubber were provided by Dujiangyan Huayi Rubber Co., Ltd., (Chengdu, China), and are listed in Table 1. The coarse aggregates (CAs) are selected from crushed granite, with a particle size range of 5–16 mm. According to the standard GB/T 14685-2011 [31], the properties of coarse aggregates were obtained from tests, as shown in Table 1. The particle size distribution of sand, recycled rubber, and coarse aggregates are shown in Figure 2. The SFs used in this study are straight and copper plated. The parameters of SFs are provided by the supplier Henan Zange Industrial Co., Ltd., (Zhengzhou, China), as shown in Table 2. Two varieties of alkali-resistant GFs with fiber lengths of 6 mm and 12 mm were employed in order to investigate the impact of GF length on the compressive behavior of RRC. The only difference between the two kinds of GFs is their length. The supplier Taishan Glass Fiber Co., Ltd., (Taian, China) provides the GFs’ qualities, which are displayed in Table 2. The superplasticizer (SP), which utilizes an admixture based on polycarboxylates, has a specific gravity of 1.02 and a solid content of 9%. The dosage of SP is 0.5% of the cement mass.

### 2.2. Design of Concrete Mix

In the concrete mix design, four SF contents (0, 0.4%, 0.8%, and 1.2%), four GF contents (0, 0.2%, 0.4%, and 0.6%), and two GF lengths (6 mm and 12 mm) were employed to examine the combined impacts of SFs and GFs on the workability and compressive behavior of RRC. A proportion of the concrete’s volume was used to represent the fiber content. Based on current research, the rubber content was approximately 10%, and its negative influence on the mechanical properties of concrete was slight; therefore, the recycled rubber content in this study was taken as 10%. The recycled rubber content was expressed as a percentage of the fine aggregate volume. The SP dosage was expressed as a percentage of the mass of cement, which was fixed at 0.5% in this study. The ratio of cement to water was set at 0.4. A total of 28 RRC mixes in all were created, as shown in Table 3. The S-G-L formula was used to label each concrete mix. S is S0, S0.4, S0.8, or S1.2, representing SF contents of 0, 0.4, 0.8, or 1.2%, respectively. G is either G0, G0.2, G0.4, or G0.6, representing GF contents of 0, 0.2, 0.4, or 0.6%, respectively. Similarly, L was either L6 or L12, representing GF lengths of 6 or 12 mm, respectively.

### 2.3. Design of Concrete Specimens

Three examples for each concrete mix, for a total of eighty-four specimens, were created for the compressive testing, and their measurements were Φ100 × 200 mm. Three specimens were prepared for each concrete mix, for a total of eighty-four specimens. The following three steps were taken to uniformly distribute the fibers in the concrete. (1) First, cement, sand, rubber, and fibers were poured into the mixer and stirred for 60 s to ensure uniform mixing; (2) after thoroughly mixing the water and SP, approximately 70% of the solution was added to the mixer and stirred for 60 s; and (3) CAs were added, and the remaining 30% of the mixed solution was added to the mixer and stirred for 180 s until it was thoroughly mixed. According to the standard GB/T 50080-2016 [32], the workability of the fresh concrete was tested. Then, the fresh concrete was poured into the prepared plastic mold. Three specimens were prepared for each mix. The samples were then cured at room temperature (23 °C) for 24 h. After one day of curing, the specimens were demolded and stored in water for 28 d and then removed and wiped dry for the compressive tests.

### 2.4. Test Setup and Method

**Figure 3:**
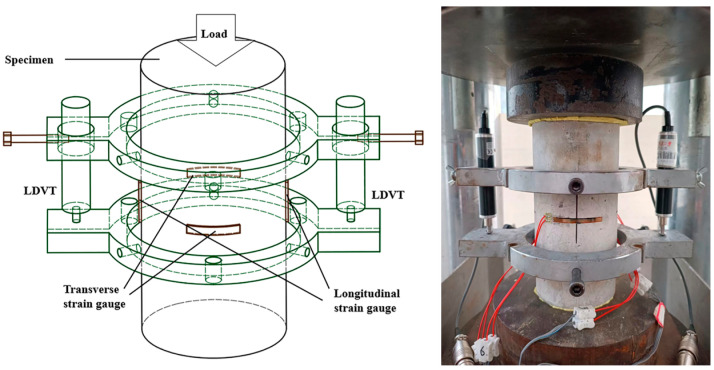
Compressive test configuration.

The settings for the axial compression tests are shown in Figure 3. Plaster was applied before loading to ensure the flatness and parallelism of the upper and lower surfaces of the specimen under compression. At the specimen’s mid-height, two 50 mm long longitudinal strain gauges were symmetrically adhered. Likewise, two symmetrically glued 50 mm long transverse strain gauges were attached to the specimen’s midpoint. Two linear variable differential transducers (LVDTs) were set up within an 80 mm length at the specimen’s mid-height to measure the axial strain because the strain gauges were destroyed after the concrete cracked. Based on the standard ASTM C39/C39M [33], the experimental loading adopted a displacement control mechanism with a loading rate of 0.18 mm/min. Before the formal loading, the specimen was preloaded and centered according to the readings of the strain and displacement gauges. The specimen was subjected to axial compression before it was subjected to formal loading. The load and deformation values during the loading process were recorded. The axial deformations of the specimens were measured using linear variable differential transformers and a longitudinal strain gauge. Similarly, a transverse strain gauge was used to quantify the specimens’ transverse deformations. Equations (1) and (2) may be used to compute the axial stress *σ* and strain *ε* of the specimens during the loading process based on the collected data.

(1) σ=P/A

(2) ε=s/80

where *P* denotes the applied load, *A* denotes the specimen’s cross-sectional area, *s* denotes the displacement as measured by the LVDTs (in mm), and 80 is the LVDTs’ gauge length (in mm).

## 3. Slumps and Failure Mode

### 3.1. Slumps

**Figure 4:**
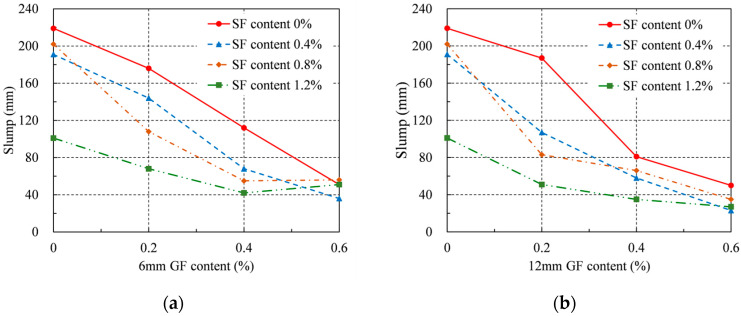
Slump: (**a**) 6 mm GF; (**b**) 12 mm GF.

Table 4 and Figure 4 show the fresh RRC slumps. The slump was reduced to 101–202 mm when just SFs were added. The slump shows a decreasing trend as SF content increases, which is similar to the conclusion in the literature [34]. This is because SFs are prone to cross-overlapping, forming a grid structure and supporting the concrete. The slump was reduced to 51–176 mm when just 6 mm of GFs were added. The slump was reduced to 50–187 mm when just 12 mm GFs were added. Because the fibers can restrict the flow of fresh RRC, adding more fibers greatly reduces the slump for the same GF length [35]. The decline was unaffected by GF length for the same GF content. Moreover, by comparing the slumps using single fibers, it was observed that the impact of GFs on the slump was more apparent than the slumps using single fibers. This is because, under the same volume content, the quantity of GFs is greater than SFs and has a larger total surface area. This leads to an increase in the amount of paste required to encase the fibers and a decrease in the amount of paste between the aggregates. Macroscopically, this manifests as a decrease in the workability of fresh RRC. Worse workability increases the porosity of concrete and degrades its mechanical properties.

### 3.2. Failure Modes and Mechanisms

**Figure 5:**
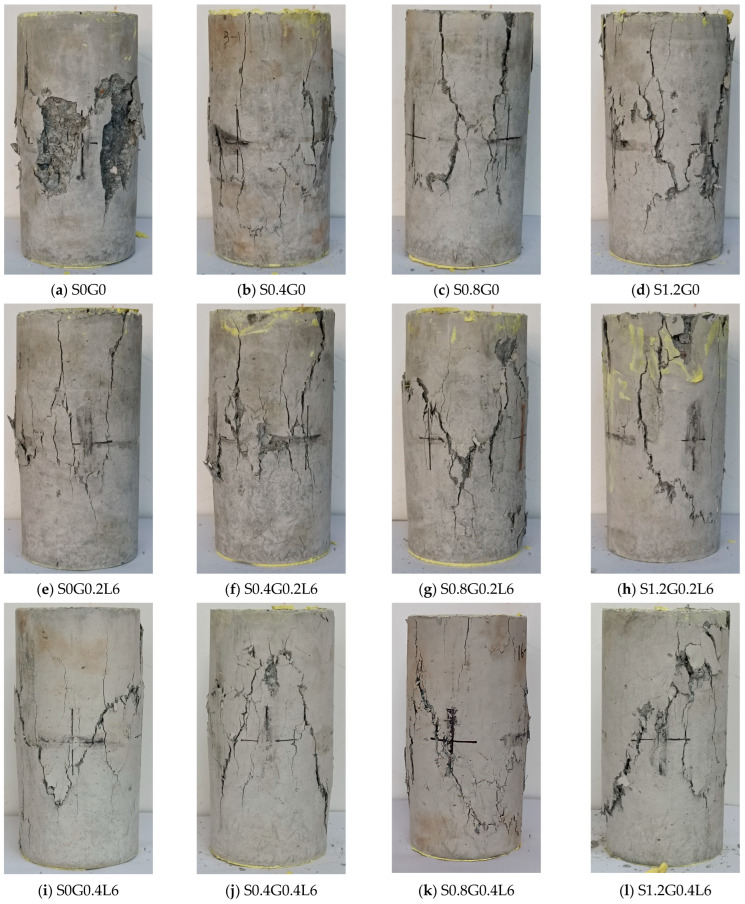

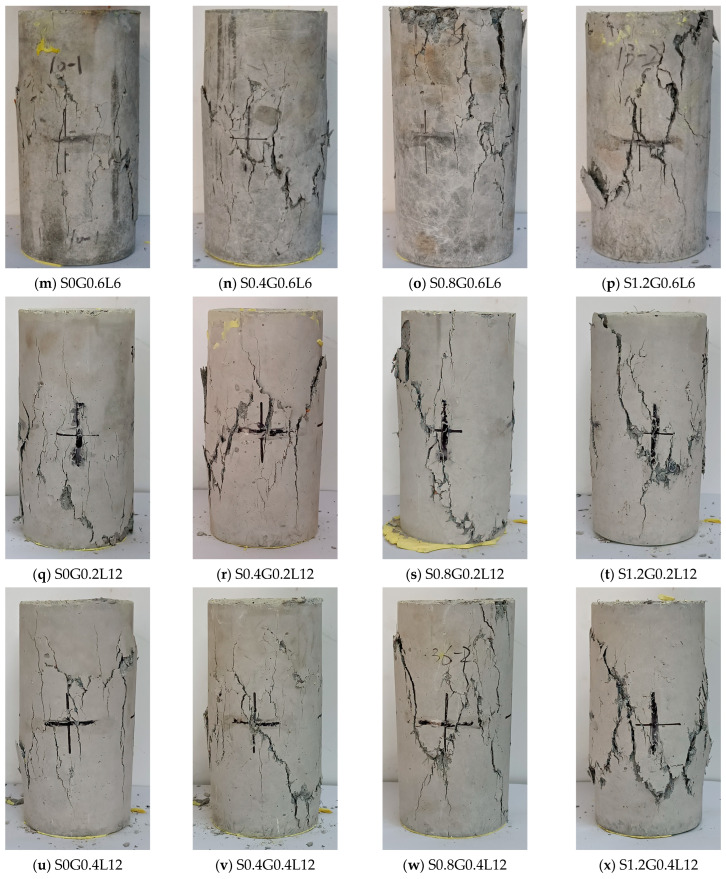

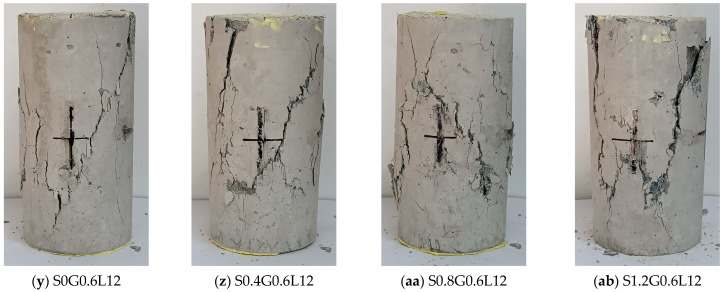
Failure modes of HFRRRC.

The failure modes of the specimens after the axial compressive tests are shown in Figure 5. For the specimen without fibers (corresponding to Figure 5a), vertical cracks first appeared in the mid-high region of the specimen. At both ends of the specimen, cracks appeared as the load increased. After the load reached its peak, cracks started to form at the specimen’s ends and progressed toward the middle until they met the central cracks. At the end of the test, the concrete specimen developed significant cracks, and the blocky concrete began to peel away from its surface.

In RRC compressive specimens with only SFs (corresponding to Figure 5b–d), the SFs formed a skeleton inside the concrete, effectively resisting deformation in the load direction and constraining transverse deformation. At the initial loading, the deformation of the cylinder was relatively small, and no cracks appeared. As the load approached its peak value, vertical cracks appeared at the mid-height of the specimen. After the specimen reached its peak load, the width of the cracks increased further; however, no pieces fell off when the specimen failed. As the SF content increased, the final crack width increased. This is because the SFs significantly improved the ductility of RRC. The bearing capacities of the specimens decreased slowly after reaching the peak load, resulting in a larger loading displacement when the concrete failed completely.

For RRC compressive specimens with only GFs (corresponding to Figure 5e,i,m,q,u,y), longitudinal deformation occurred after the initial loading, accompanied by obvious transverse deformation. As the load approached the peak value, significant cracks appeared at the mid-height of the specimen and developed toward both ends along the direction parallel to the load. Later, when the deformation intensified, diagonal cracks started to grow at the specimens’ ends and progressed until they met up with the middle cracks. Finally, after the peak load was reached, cracks developed quickly until the specimen failed. As the GF content increased, the crack propagation rate of the specimens decreased after the peak load. As a result, the GFs slightly improved the ductility of RRC, but as the GF content increased, the surface of the specimen developed finer cracks.

For RRC compressive specimens with hybrid fibers (corresponding to the remaining images in Figure 5), the failure modes combined the characteristics of the two types of single-fiber RRC. With an increase in the SF content, the concrete exhibited a stronger resistance to deformation, and cracks appeared later. At the same time, the SFs considerably increased RRC’s ductility after the peak load, increasing the loading displacement for specimen failure and forming a wider crack. With an increase in GF content, GFs effectively restricted the cracks to fine cracks, and a larger number of fine cracks were formed. Therefore, the specimen absorbed more energy when the concrete failed. As the GF content increased, GFs effectively limited the cracks to fine cracks, and more fine cracks occurred as a result. Therefore, the specimen absorbed more energy when the concrete failed.

## 4. Results and Discussion

### 4.1. Compressive Strength

**Figure 6:**
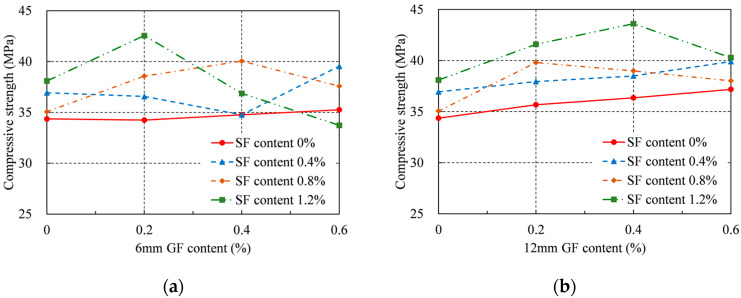
Compressive strength: (**a**) 6 mm GF; (**b**) 12 mm GF.

The compressive strengths of RRC were computed and are shown in Table 4 and Figure 6 in accordance with ASTM C39/C39M [33]. As the GF content increased, the compressive strength of each series initially increased and then decreased. This indicated that more GFs were not necessarily preferred. Therefore, each set of specimens had the ideal GF content. The addition of GFs of both lengths enhanced the compressive strength of the series without SFs. When the series contained 0.6% GF, for the 6 mm GF and the 12 mm GF, the highest improvement in compressive strength was 3% and 8%, respectively. When the series contained SFs, when the 6 mm GF content was 0.2% and the SF content was 1.2%, the greatest increase in compressive strength was around 24%, and when the 12 mm GF content was 0.4% and the SF content was 1.2%, the maximum increase in compressive strength was around 27%. As the SF content increased, the optimal GF content in each series decreased. This is because a large number of fibers causes the concrete flowability to decrease significantly, thereby increasing the porosity of the concrete. The optimal content for the 6 mm GF was 0.2%, while the optimal content for the 12 mm GF was 0.4%. Overall, the effect of the 12 mm GF on improving the compressive strength was more significant.

Additionally, when only the SFs were introduced at 1.2%, the greatest gain in compressive strength was 11%. Similar to this, adding merely 12 mm GFs at 0.6% increased compressive strength by a maximum of 8%. However, the largest gain in compressive strength for the 12 mm GFs at a content of 0.4% and SFs at a content of 1.2% occurred when both GFs and SFs were applied. Therefore, it can be said that GFs and SFs both increase the compressive strength of RRC when added independently. However, the largest compressive strength was achieved when GFs and SFs were added together. S1.2G0.4L12 had the highest compressive strength, and the corresponding single-fiber specimens were S1.2G0 and S0G0.4L12. The failure modes are shown in Figure 5d,u,x. This hybrid effect is caused by the distinct characteristics of the two types of fibers. Due to the high strength and elastic modulus of the SF, it can form a skeleton inside the concrete and provide bridging effects across cracks at the initial stages of cracking. The SFs were removed and failed when the cracks enlarged. In addition, after the addition of GFs with larger aspect ratios, more small cracks appeared when the concrete was damaged. This is because a large number of GFs can effectively reduce the degree of stress concentration at the crack tip and redistribute the stress inside the concrete. GFs allow more matrix materials to function, thereby improving the strength and toughness of RRC.

Compressive strength did not, however, typically rise with increasing GF and SF contents. To put it another way, there are ideal GF and SF contents to provide the greatest compressive strength. The ideal range for total fiber content was 1.4% to 1.6%. Excessive fiber content can severely affect the workability of RRC, leading to an increase in porosity and a decrease in compressive strength. In contrast, the optimum GF content was between 0.2% and 0.4%. Because of the greater impact on workability with equal content, the GF content needs to be strictly controlled compared with the SF.

### 4.2. Elastic Modulus

**Figure 7:**
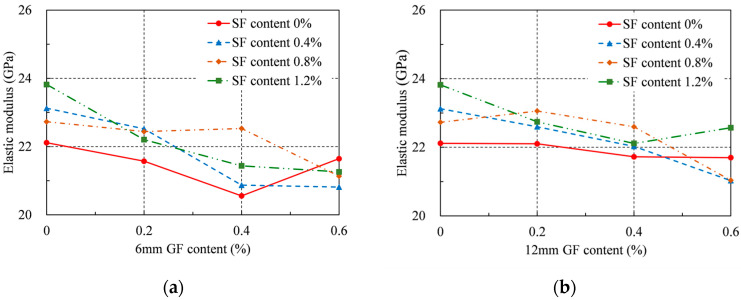
Elastic modulus: (**a**) 6 mm GF; (**b**) 12 mm GF.

The elastic modulus of RRC was computed and shown in Table 4 and Figure 7 in accordance with ASTM C469/C469M [36]. As the GF content grew, the elastic modulus of each series decreased, which is similar to the conclusion in the literature [37]. For each series, the maximum elastic modulus occurred when the GF content was 0%, except for the series with 0.8% SFs. Specifically, for the series with 0.8% SFs, the elastic modulus growth rates were found to be very close; that is, 3, 1, and 2% when the 6 mm GF content was 0, 0.2%, and 0.4%, respectively. The elastic modulus increased by a maximum of 4% for the 0.8% SF series when the 12 mm GFs content was 0.2%. The addition of the GFs had a passive effect on the elastic modulus. However, an improvement was observed when SFs were added, particularly at concentrations of 0.8%.

Furthermore, when merely SFs were supplied at a 1.2% rate, the elastic modulus could rise a maximum of 8%. The elastic modulus of RRC dropped to varied degrees when just GFs were introduced. However, when both GFs and SFs were applied, the largest gain in the elastic modulus was 4% for the 12 mm GFs at a content of 0.2% and SFs at a content of 0.8%. Therefore, even though hybrid fibers can somewhat increase the elastic modulus of RRC, the improvement is not as notable as that obtained by only SF addition. After the addition of GFs, the workability of RRC decreased, resulting in a larger porosity inside RRC. This significantly reduces the ability of the concrete to resist deformation and causes the elastic modulus to decrease. SFs have high stiffness and can form a skeleton inside the concrete to improve its ability to resist deformation. The positive impact of increasing the SF content on the elastic modulus significantly outweighed its negative impact on the workability of RRC. Therefore, when only SFs were added, the elastic modulus reached its maximum value at an SF content of 1.2%. As shown in Figure 4, when the total fiber content was greater than 1%, the working performance of the concrete significantly decreased. Therefore, the ideal elastic modulus for the hybrid fiber series was obtained at a 12 mm GF content of 0.2% and an SF content of 0.8%, respectively.

### 4.3. Poisson’s Ratio

**Figure 8:**
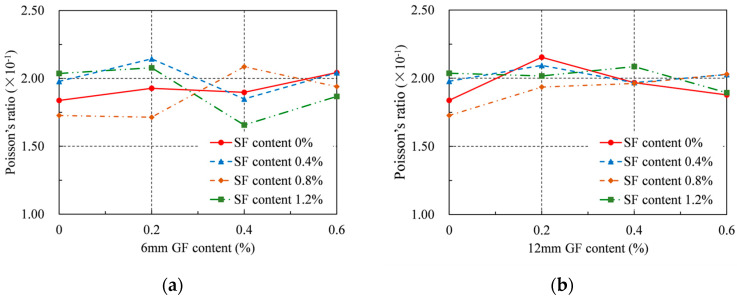
Poisson’s ratio: (**a**) 6 mm GF; (**b**) 12 mm GF.

Poisson’s ratio of RRC was determined and shown in Table 4 and Figure 8 in accordance with ASTM C469/C469M [36]. For the series adding 6 mm GFs, Poisson’s ratio of each series shows different patterns as the GF content increases, but overall, it is within the range of 0.166–0.214 or 0.190 ± 0.024. For the series with 12 mm GFs, Poisson’s ratio of each series initially grew and then declined as the GF content increased, with the exception of the series with 0.8% SFs, whose Poisson’s ratio climbed constantly as the GF content increased. For the series with 12 mm GFs, Poisson’s ratio was within the range of 0.173–0.215 or 0.194 ± 0.021. Because of this, the effects of the GFs and SFs on Poisson’s ratio in this experiment are rather minimal. At the same time, the ranges of Poisson’s ratio corresponding to 6 mm and 12 mm GFs are very close, indicating that the length of the GFs has little effect on Poisson’s ratio.

### 4.4. Strain at Peak Stress

**Figure 9:**
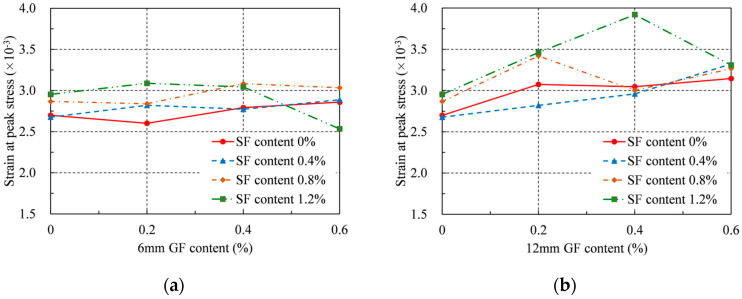
Strain at peak stress: (**a**) 6 mm GF; (**b**) 12 mm GF.

**Figure 10:**
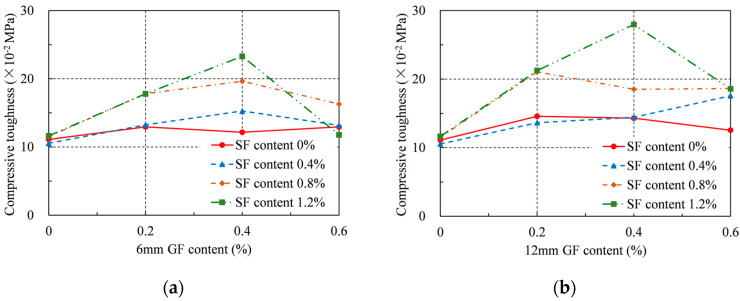
Compressive toughness: (**a**) 6 mm GF; (**b**) 12 mm GF.

The strain at the peak stress is defined as the average longitudinal strain within the middle 80 mm range of a concrete specimen when it reaches compressive strength, as shown in Table 4 and Figure 9. The impact of the GF content on the strain at the peak stress differed for various RRC series. The strain at peak stress rose with an increase in the 6 mm GF content for the series without SFs, but it increased monotonically with an increase in the 12 mm GF content. When the GF content was 0.6%, both GF lengths produced the maximum increase in strain at peak stress. These values were 6% and 17% for the 6 mm and 12 mm GFs, respectively. For the series containing SFs, when the 6 mm GFs content was 0.2% and the SFs content was 1.2%, the maximum increase in strain at peak stress was roughly 14%, and when the 12 mm GFs content was 0.4% and the SFs content was 1.2%, the maximum increase in strain at peak stress was roughly 45%. Moreover, by comparing Figure 10a,b, it can be concluded that the 12 mm GFs had a more significant impact on the strain at peak stress.

When only the SFs were introduced at a 1.2% rate, the highest strain increase at peak stress was 9%. Similarly, when only 12 mm GFs were introduced at 0.6%, the highest increase in strain at peak stress was 17%. The largest increase in strain at peak stress, however, was 45% for the 12 mm GFs at a content of 0.4% and 1.2% for the SFs when both GFs and SFs were introduced. As a result, it can be said that the addition of the GFs and SFs independently enhanced the strain at RRC’s peak stress. However, the strain at peak stress was the largest when GFs and SFs were added together. Similar to compressive strength, there is a synergistic impact on the strain at peak stress.

The strain at the peak stress did not, however, invariably rise with rising GF and SF content. To put it another way, the GF and SF content had ideal values for attaining the greatest strain at the peak stress. Overall, the positive effect of GF on the peak strain was limited when its content exceeded 0.4%. In particular, in the series with SF contents greater than 0.8%, excessive amounts of GFs may even have a negative effect on the strain at peak stress. Therefore, to improve the strain at the peak stress of RRC, the GF content should not exceed 0.4%, and the total fiber content should lie between 1.4% and 1.6%.

### 4.5. Compressive Toughness

Compressive toughness was calculated according to the following Equation (3), which reflects the energy absorption capacity of RRC under compressive loads:

(3) $$T=\int_{0}^{\varepsilon_{0.8}}\sigma d\varepsilon$$

where *T* is the compressive toughness and *ε*_0.8_ is the strain when the load is equal to 80% of the peak load after the peak point. The calculated compressive toughness is presented in Table 4 and Figure 10. For the specimens without SFs, the compressive toughness first increased and then decreased with increasing GF content. The greatest improvement in compressive toughness at 0.2% GF concentration was 16% for 6 mm GF and 31% for 12 mm GF. For the series with added SFs, the greatest improvement in compressive toughness was 110% for the 6 mm GF and 152% for the 12 mm GF, respectively, when the GF content was 0.4% and the SF content was 1.2%.

Additionally, the SFs introduced at 1.2% resulted in a 5% maximum improvement in compressive toughness. Similarly, when only 12 mm GFs were added at 0.2%, the greatest gain in compressive toughness was 31%. However, the largest improvement in compressive toughness was 152% for the 12 mm GFs at a content of 0.4% and SFs at a content of 1.2% when both GFs and SFs were applied. Therefore, it can be said that the mixed addition of GFs and SFs can better improve the compressive toughness of RRC. Because of their larger quantity and smaller density compared with SFs, GFs can be distributed more uniformly inside the concrete, allowing more matrix materials to function, thereby improving the compressive toughness of RRC. This was confirmed by the failure mode of RRC, where the specimens with added GFs had more fine cracks. The presence of SFs can prevent the rapid development of large cracks, thereby preventing GFs from being pulled out and failing early. At the same time, longer fibers can provide a greater bridging effect. Hence, the 12 mm GF is better than the 6 mm for improving the compressive toughness. However, to improve toughness, the GF content should not exceed 0.4%.

## 5. Mechanism Analysis

The compressive strength, elastic modulus, strain at peak stress, and compressive toughness of RRC were all somewhat enhanced by the addition of SFs. The three primary components of the SFs’ influence on the compressive behavior of RRC are as follows. (1) Because of the high strength and high elastic modulus of SFs, they can effectively control the macroscopic cracks in RRC under compressive tests, thereby improving the compressive behavior of RRC. (2) The rigid SFs can form a skeleton inside the concrete, thereby improving the ability of RRC to resist deformation. (3) SFs have a negative effect on the workability of fresh RRC, which leads to an increase in the internal porosity of RRC, reducing its compressive behavior. In this study, the effect of the SFs on the workability of RRC was relatively small; therefore, the comprehensive effect of the SFs was usually positive.

The addition of GFs can greatly increase the strain at peak stress and the compressive toughness of RRC while only modestly enhancing the compressive strength of RRC. However, GFs have the ability to lower RRC’s elastic modulus. The three primary components of the GFs’ influencing mechanism on the compressive behavior of RRC are as follows. (1) Because of the larger aspect ratio of GFs, the number of GFs is far greater than SFs under the same volume fraction. The GFs can effectively suppress the development of microcracks inside RRC under a compression load, thereby slowing down the process of reaching the peak stress of RRC. (2) The GFs uniformly distributed in RRC can effectively reduce the stress concentration at the crack tip and redistribute the stress inside RRC. GFs allow more matrix concrete to function, thereby improving the compressive toughness of RRC. (3) GFs have a significant negative effect on the workability of fresh RRC, which leads to a decrease in the compressive behavior of RRC. Under the combined influence of these negative effects and the aforementioned positive effects, an optimal GF content often exists.

The compressive strength, strain at peak stress, and compressive toughness were all significantly improved by the mixed addition of SFs and GFs, while the elastic modulus was improved the most when just SFs were added. The synergistic impact of both fibers is the primary cause of the improvement in HFRRRC’s compressive behavior. (1) In the initial stage of the experiment, the GFs can effectively inhibit the generation and development of microcracks. (2) When microcracks develop into macroscopic cracks, the presence of SFs can prevent the rapid development of macroscopic cracks, avoid brittle failure, and prevent the premature extraction or fracture failure of GFs. (3) The bonding between the 12 mm GFs and RRC was better; therefore, the optimal values of various indicators usually appeared in the 12 mm GFs test group.

In general, adding either SFs or GFs can slightly increase the compressive strength of RRC, however, the compressive behavior can be most effectively improved by adding both fibers. This indicates that there is a positive synergistic effect between the two types of fibers on the compressive behavior of RRC. Variations in the experimental results, however, are complicated since the fiber content has a major influence on the workability of fresh RRC. The mechanism of action has to be clarified by more studies.

## 6. Constitutive Analysis

### 6.1. Stress–Strain Curves

**Figure 11:**
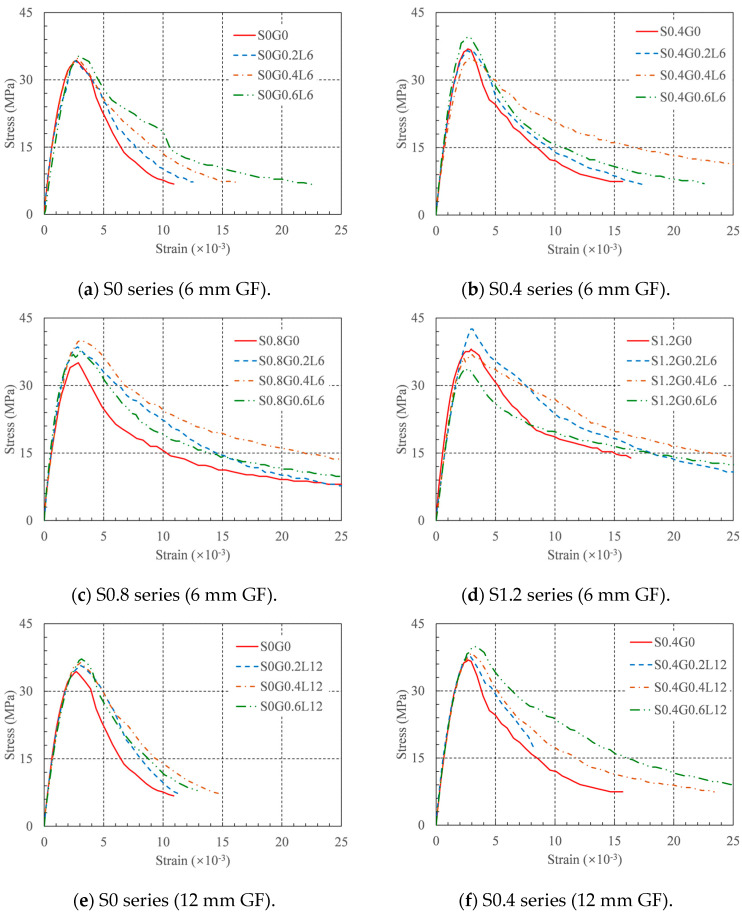

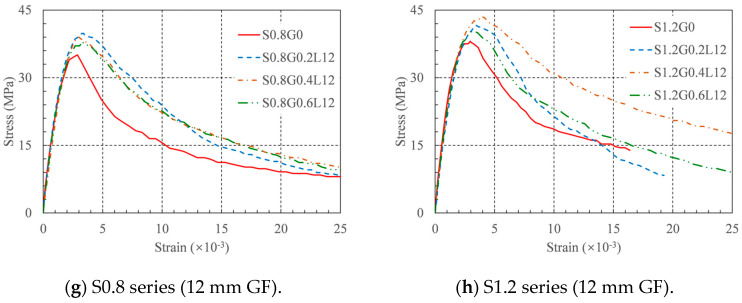
Stress–strain curves.

The stress–strain curves for each concrete mixture are shown in Figure 11. In Figure 11, it can be observed that all stress–strain curves can be divided into three stages: the linear rise (from the beginning of loading to 40% compressive strength), nonlinear rise (from the proportional limit point to the compressive strength), and decline (from the compressive strength to the failure point of the specimen). The stress–strain curve of RRC’s form and properties were significantly influenced by both the SFs and GFs. In the following sections, a constitutive relationship is established based on the experimental data to predict the stress–strain relationship of HFRRRC.

### 6.2. Constitutive Model

First, the constitutive model shown below was utilized to accurately reproduce the form properties of the experimental stress–strain curve when fitting the experimental data:

(4) $${\sigma =f}_{c}\left[a\frac{\varepsilon }{\varepsilon_{c}}+\left(3-2a\right){\left(\frac{\varepsilon }{\varepsilon_{c}}\right)}^{2}+\left(a-2\right){\left(\frac{\varepsilon }{\varepsilon_{c}}\right)}^{3}\right]\;({\varepsilon <\varepsilon }_{c})$$

(5) $$\sigma =f_{c}\frac{\frac{\varepsilon }{\varepsilon_{c}}}{b{\left(\frac{\varepsilon }{\varepsilon_{c}}-1\right)}^{2}+\frac{\varepsilon }{\varepsilon_{c}}}\;(\varepsilon \geq \varepsilon_{c})$$

where *f_c_* is the compressive strength, *ε_c_* is the strain at peak stress, and *a* and *b* are the controlling parameters. Equations (4) and (5) were used to fit the rising and descending segments of the stress–strain curve, respectively [38,39]. By fitting the test stress–strain curve, the values of the above two controlling parameters were obtained, as shown in the last two columns of Table 4. All the values of R2 were greater than 0.932. This indicates that the constitutive model can accurately predict the stress–strain curve of HFRRRC.

Second, as changes in the material parameters of the fibers will significantly affect the compressive behavior of RRC, the reinforcement index (*RI*) of the fibers was calculated according to the following equations:

(6) $${\mathrm{RI}}_{S}{=V}_{S}\frac{L_{S}}{d_{S}}$$

(7) $${\mathrm{RI}}_{G}{=V}_{G}\frac{L_{G}}{d_{G}}$$

where *RI_S_* and *RI_G_* represent the reinforcement indices of the SFs and GFs, respectively, vs. and *V_G_* represent the volume fractions of the SFs and GFs, respectively, *L_S_* and *L_G_* represent the SFs and GFs lengths, respectively, and *d_S_* and *d_G_* represent the diameters of the SFs and GFs, respectively.

Finally, using a regression analysis based on the following polynomial formula, the compressive strength *f_c_*, strain at peak stress *ε_c_*, controlling parameter *a*, and controlling parameter *b* of RRC were associated with RIS and RIG.

(8) $$F_{i}{=\alpha }_{i1}{\mathrm{RI}}_{S}{}^{3}{+\alpha }_{i2}{\mathrm{RI}}_{G}{}^{3}{+\alpha }_{i3}{\mathrm{RI}}_{S}{}^{2}{\mathrm{RI}}_{G}{+\alpha }_{i4}{\mathrm{RI}}_{S}{\mathrm{RI}}_{G}{}^{2}{+\alpha }_{i5}{\mathrm{RI}}_{S}{}^{2}{+\alpha }_{i6}{\mathrm{RI}}_{G}{}^{2}{+\alpha }_{i7}{\mathrm{RI}}_{S}{\mathrm{RI}}_{G}{+\alpha }_{i8}{\mathrm{RI}}_{S}{+\alpha }_{i9}{\mathrm{RI}}_{S}{+\alpha }_{i10}$$

where, *F_i_* (*i* = 1, 2, 3, 4) represents the compressive strength, strain at peak stress, controlling parameters *a* and *b*, respectively, and *α_ij_* (*i* = 1, 2, 3, 4; j = 1, 2, …, 10) is the coefficient determined by regression analysis for each indicator, and the results of each coefficient are shown in Table 5. It is worth noting that, owing to regression analysis on the data obtained with the 6 mm and 12 mm GFs, a set of coefficients *α_ij_* was independently determined for each GF length. In Table 6, the expected outcomes for the compressive strength and strain at the peak stress are shown. The predicted values of the compressive strength were within 6% of the experimental values, whereas the predicted values of the strain at peak stress were within 10% of the experimental values. As a result, using this formula, it is possible to accurately forecast the compressive strength and strain at the peak stress of HFRRRC.

**Figure 12:**
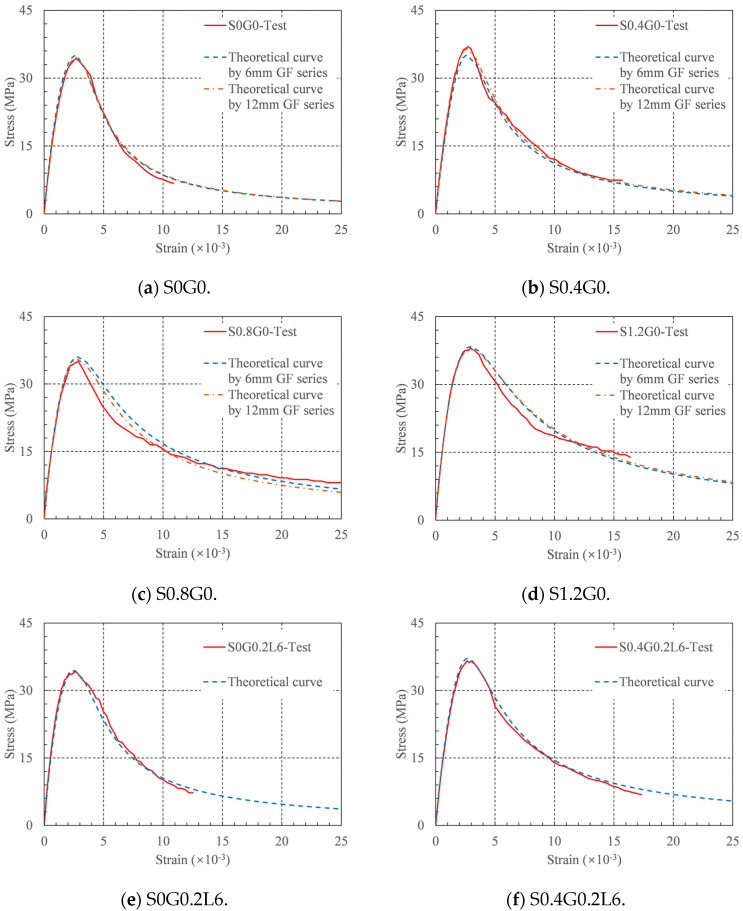

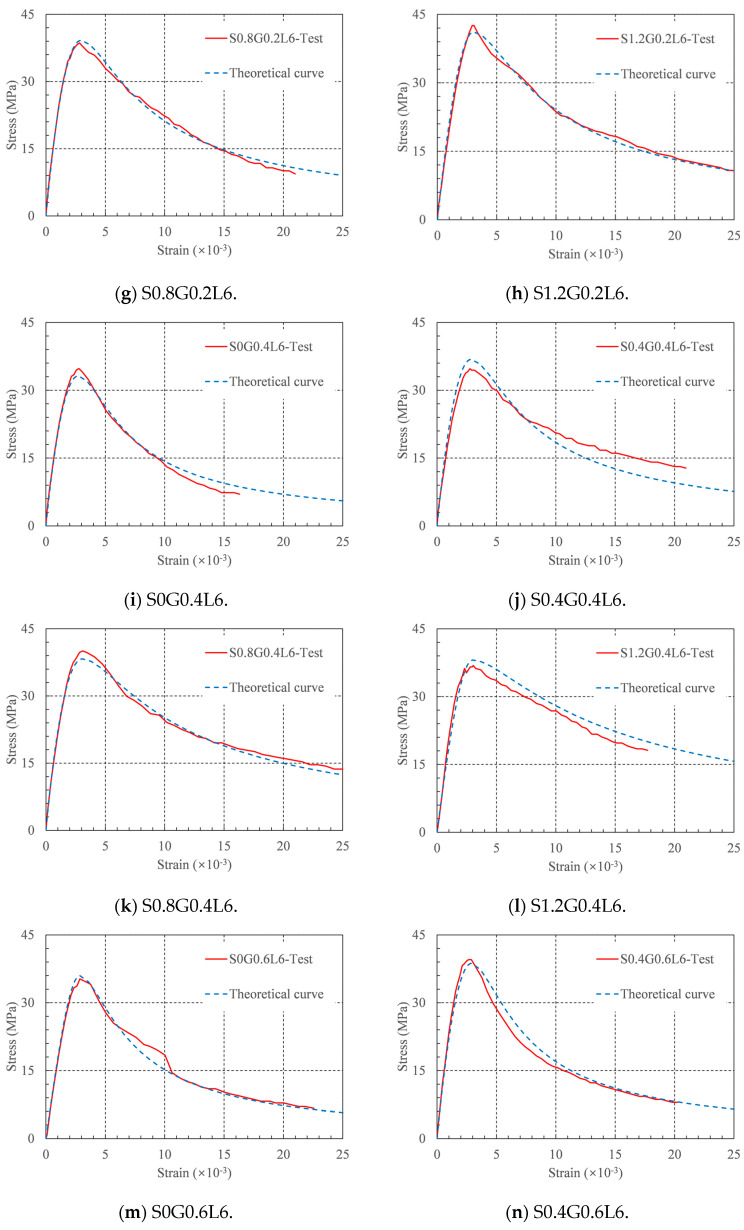

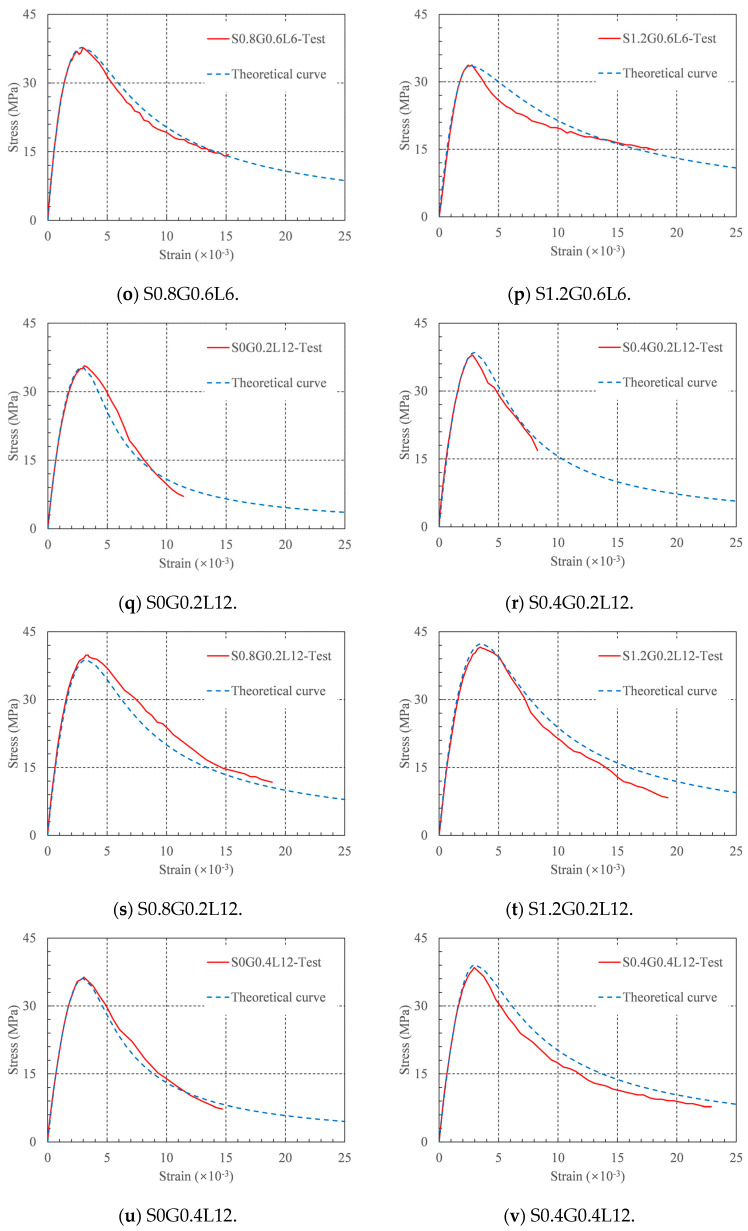

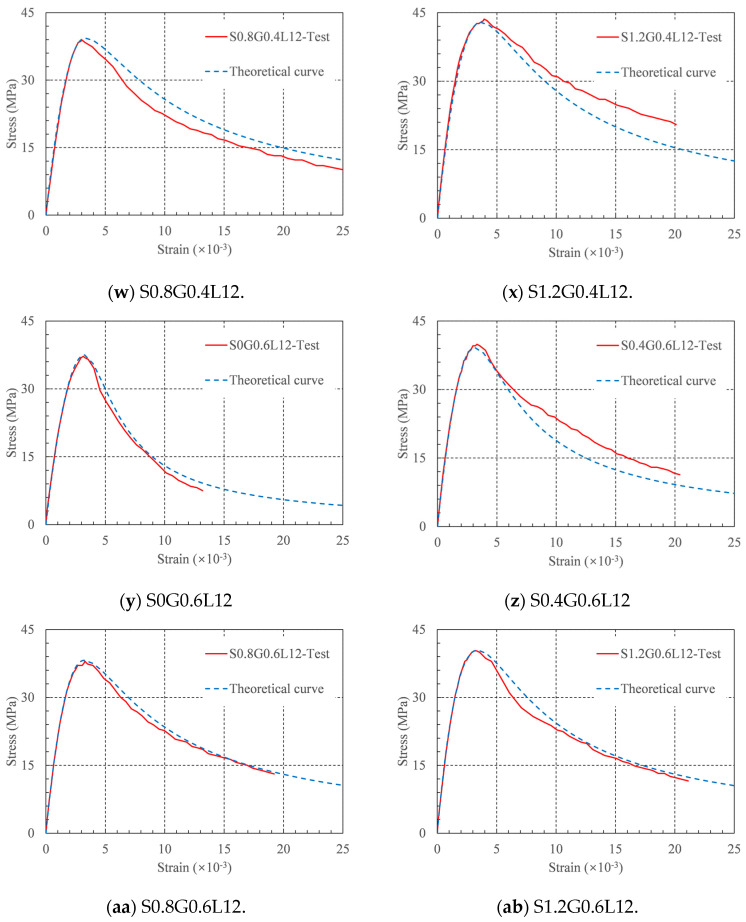
Stress–strain curves in theory and experiment.

The coefficients in Table 5 can be used to construct the axial compressive stress–strain constitutive models of RRC with different fiber contents. The generated constitutive model and the experimental stress–strain curves are compared in Figure 12. Through comparison, it can be found that the theoretical curve is in good agreement with the experimental curve, indicating that the proposed constitutive model can accurately predict the stress–strain curve of HFRRRC. This is because the mechanism of how SFs and GFs affect the compressive behavior of RRC is complicated. In this study, the RI of the two types of fibers was fitted as a variable simultaneously, which led to a decline in the goodness of fit. A large amount of experimental research is required to fully investigate the failure mechanism and constitutive model of the HFRRRC. However, our investigation showed that Equations (4) and (5) may accurately forecast the stress–strain curve of this novel concrete. The suggested model can simultaneously forecast the stress–strain curve of HFRRRC with any fiber composition.

## 7. Conclusions

To study the influence of SFs and GFs on the compressive behavior of RRC, 28 concrete mixes were designed, and axial compression tests were conducted. From the experimental results, the following conclusions were drawn:

(1) An increase in the SF and GF contents can deteriorate the workability of fresh RRC, and the GF content has a more significant impact on the workability of fresh RRC. For the same fiber content, the length of the GFs had a slight effect on the workability of fresh RRC.
(2) SF can effectively improve the deformation resistance of RRC, delay the generation of cracks, and increase the ductility of RRC after the peak load. The GF can effectively suppress the development of microcracks into large cracks, resulting in more small cracks on the surface of the concrete specimens while increasing the energy absorbed by RRC during failure.
(3) Single or hybrid fibers can improve the compressive behavior of RRC. The addition of fibers resulted in maximum growth rates of 27%, 8%, 45%, and 152% for the compressive strength, elastic modulus, strain at peak stress, and compressive toughness, respectively. At the same time, Poisson’s ratio ranged from 0.166 to 0.215. These positive effects are attributed to the bridging effect of SFs and GFs after the generation of cracks, which can effectively suppress the generation and development of macrocracks and microcracks.
(4) The improvement of the compressive behavior of RRC due to 12 mm GF was more significant than 6 mm GF. Specifically, in this study, the optimal compressive strength, strain at peak stress, and compressive toughness were obtained with hybrid 12 mm GF and SF. Owing to the larger aspect ratio of the 12 mm GF, the improvement in the compressive behavior of RRC is greater than the 6 mm GF.
(5) There are optimal SF and GF contents that improve the compressive behavior of RRC. For compressive strength, the optimal total fiber content ranges from 1.4% to 1.6%. The optimal GF content was between 0.2% and 0.4%. When the GF content is greater than 0.4%, it tends to reduce the compressive behavior of RRC; therefore, it is recommended that the GF content be less than 0.4%.
(6) A constitutive model for the HFRRRC was constructed based on experimental data. The coefficients of compressive strength, strain at peak stress, and controlling parameters (*a* and *b*) pertaining to the RIS and RIG are obtained by the model using polynomial fitting and are shown in Table 5. With varying fiber content, this model can provide stress–strain curves for RRC, and it exhibits good agreement with experimentally measured stress–strain curves. This demonstrated that the constitutive model was precise enough for real-world use.
(7) Finally, further research is needed on the following aspects of HFRRRC. Analyze the failure mechanism of the concrete through microscopic experiments and explain the macroscopic test results; test the mechanical properties under other loads, such as tensile, flexural, and impact loads; and test the durability performance, such as water resistance, frost resistance, and acid/alkali resistance.

## Figures and Tables

**Table 1:** Properties of fine and coarse aggregates.

Aggregate Type	Source	Apparent Density (kg/m^3^)	Particle Size (mm)	Water Absorption (%)	Fineness Modulus
Sand	River sand	2636	<5	0.5	2.02
Recycled rubber	Waste tires	750	<2.5	-	-
Coarse aggregate	Crushed granite	2641	5–16	2.1	-

**Table 2:** Parameters of fibers.

Materials	Length (mm)	Apparent Density (kg/m^3^)	Equivalent Diameter (μm)	Tensile Strength (MPa)	Elastic Modulus (GPa)
Steel fibers	12	7800	200	3000	200
Glass fibers	6/12	2680	14	1700	72

**Table 3:** Mix proportions of HFRRRC (kg/m^3^).

Mix Number	Cement	Water	Recycled Rubber	Sand	Coarse Aggregate	SF	GF	SP
S0G0	554.1	245.3	17.1	531.2	966.3	0.0	0.0	2.8
S0.4G0	551.9	244.4	17.0	529.1	962.4	31.2	0.0	2.8
S0.8G0	549.7	243.4	16.9	527.0	958.5	62.4	0.0	2.8
S1.2G0	547.5	242.4	16.9	524.8	954.7	93.6	0.0	2.7
S0G0.2L6	553.0	244.9	17.0	530.2	964.3	0.0	5.4	2.8
S0.4G0.2L6	550.8	243.9	17.0	528.0	960.5	31.2	5.4	2.8
S0.8G0.2L6	548.6	242.9	16.9	525.9	956.6	62.4	5.4	2.7
S1.2G0.2L6	546.4	241.9	16.8	523.8	952.7	93.6	5.4	2.7
S0G0.4L6	551.9	244.4	17.0	529.1	962.4	0.0	10.7	2.8
S0.4G0.4L6	549.7	243.4	16.9	527.0	958.5	31.2	10.7	2.8
S0.8G0.4L6	547.5	242.4	16.9	524.8	954.7	62.4	10.7	2.7
S1.2G0.4L6	545.2	241.4	16.8	522.7	950.8	93.6	10.7	2.7
S0G0.6L6	550.8	243.9	17.0	528.0	960.5	0.0	16.1	2.8
S0.4G0.6L6	548.6	242.9	16.9	525.9	956.6	31.2	16.1	2.7
S0.8G0.6L6	546.4	241.9	16.8	523.8	952.7	62.4	16.1	2.7
S1.2G0.6L6	544.1	240.9	16.7	521.7	948.9	93.6	16.1	2.7
S0G0.2L12	553.0	244.9	17.0	530.2	964.3	0.0	5.4	2.8
S0.4G0.2L12	550.8	243.9	17.0	528.0	960.5	31.2	5.4	2.8
S0.8G0.2L12	548.6	242.9	16.9	525.9	956.6	62.4	5.4	2.7
S1.2G0.2L12	546.4	241.9	16.8	523.8	952.7	93.6	5.4	2.7
S0G0.4L12	551.9	244.4	17.0	529.1	962.4	0.0	10.7	2.8
S0.4G0.4L12	549.7	243.4	16.9	527.0	958.5	31.2	10.7	2.8
S0.8G0.4L12	547.5	242.4	16.9	524.8	954.7	62.4	10.7	2.7
S1.2G0.4L12	545.2	241.4	16.8	522.7	950.8	93.6	10.7	2.7
S0G0.6L12	550.8	243.9	17.0	528.0	960.5	0.0	16.1	2.8
S0.4G0.6L12	548.6	242.9	16.9	525.9	956.6	31.2	16.1	2.7
S0.8G0.6L12	546.4	241.9	16.8	523.8	952.7	62.4	16.1	2.7
S1.2G0.6L12	544.1	240.9	16.7	521.7	948.9	93.6	16.1	2.7

**Table 4:** Test results.

Mix Number	Slump (mm)	Compressive Strength		Elastic Modulus		Poisson’s Ratio		Strain at Peak Stress		Compressive Toughness		Controlling Parameter *a*	Controlling Parameter *b*
		RV * (MPa)	SD *	RV (GPa)	SD	RV	SD	RV (×10^−3^)	SD	RV (×10^−2^ MPa)	SD		
S0G0	219	34.36	1.54	22.11	0.91	1.84	0.05	2.70	0.16	11.11	2.20	2.11	1.58
S0.4G0	191	36.93	2.37	23.12	1.97	1.98	0.05	2.68	0.08	10.53	1.37	1.85	1.08
S0.8G0	202	35.06	1.25	22.73	1.20	1.73	0.19	2.87	0.32	11.52	1.54	2.34	0.66
S1.2G0	101	38.09	4.48	23.82	0.92	2.04	0.04	2.95	0.43	11.64	9.25	2.49	0.54
S0G0.2L6	176	34.24	1.97	21.57	1.86	1.93	0.05	2.60	0.28	12.94	5.19	2.66	1.01
S0.4G0.2L6	144	36.57	2.43	22.52	1.28	2.14	0.04	2.82	0.54	13.24	6.67	2.15	0.89
S0.8G0.2L6	108	38.57	1.79	22.44	1.15	1.71	0.04	2.84	0.24	17.82	6.06	2.14	0.48
S1.2G0.2L6	68	42.55	2.12	22.20	2.11	2.08	0.36	3.09	0.12	17.79	3.05	1.44	0.48
S0G0.4L6	112	34.77	1.01	20.56	1.12	1.90	0.06	2.79	0.13	12.17	3.49	2.08	0.89
S0.4G0.4L6	68	34.77	2.46	20.87	1.42	1.85	0.21	2.77	0.67	15.28	3.23	1.79	0.31
S0.8G0.4L6	55	40.05	1.39	22.53	1.69	2.09	0.09	3.08	0.24	19.64	1.56	2.01	0.34
S1.2G0.4L6	42	36.87	5.95	21.44	1.25	1.66	0.21	3.04	0.09	23.30	6.14	2.16	0.27
S0G0.6L6	51	35.26	2.48	21.65	1.37	2.04	0.12	2.86	0.38	12.94	4.40	1.38	0.67
S0.4G0.6L6	36	39.53	6.71	20.82	3.18	2.04	0.33	2.89	0.25	13.17	4.85	2.17	0.88
S0.8G0.6L6	56	37.58	1.43	21.13	3.35	1.94	0.13	3.03	0.24	16.29	2.29	2.74	0.52
S1.2G0.6L6	51	33.73	2.45	21.26	1.96	1.87	0.04	2.53	0.34	11.78	5.58	1.76	0.23
S0G0.2L12	187	35.68	2.10	22.10	1.89	2.15	0.10	3.07	0.53	14.58	5.75	2.25	1.33
S0.4G0.2L12	107	37.95	6.29	22.60	1.28	2.09	0.07	2.82	0.29	13.64	3.72	2.04	0.86
S0.8G0.2L12	83	39.81	1.56	23.06	2.56	1.94	0.04	3.42	0.16	21.06	3.00	2.41	0.61
S1.2G0.2L12	51	41.59	5.37	22.74	1.49	2.02	0.28	3.46	0.13	21.24	1.23	2.10	0.80
S0G0.4L12	81	36.36	3.78	21.72	2.77	1.97	0.11	3.05	0.19	14.30	3.13	2.06	1.02
S0.4G0.4L12	58	38.49	1.05	22.03	2.92	1.97	0.16	2.96	0.10	14.43	1.07	1.90	0.73
S0.8G0.4L12	66	38.99	0.98	22.60	2.21	1.96	0.08	3.00	0.15	18.51	5.44	1.68	0.44
S1.2G0.4L12	35	43.60	1.68	22.11	3.33	2.09	0.07	3.92	0.11	27.96	1.05	2.51	0.33
S0G0.6L12	50	37.18	2.03	21.70	1.21	1.88	0.13	3.15	0.11	12.55	5.70	1.93	1.42
S0.4G0.6L12	23	39.90	2.09	21.03	0.68	2.03	0.17	3.32	0.13	17.59	2.21	2.19	0.56
S0.8G0.6L12	35	38.02	4.96	21.04	2.81	2.03	0.34	3.26	0.30	18.63	3.50	2.30	0.50
S1.2G0.6L12	27	40.28	6.92	22.57	1.80	1.89	0.09	3.31	0.19	18.58	3.10	2.34	0.56

* RV: representative value; SD: standard deviation.

**Table 5:** Coefficients in the constitutive model.

Value of *j*	*α*_1*j*_ for *f_c_*		*α*_2*j*_ for *ε_c_*		*α*_3*j*_ for *a*		*α*_4*j*_ for *b*	
	6 mm GFs	12 mm GFs	6 mm GFs	12 mm GFs	6 mm GFs	12 mm GFs	6 mm GFs	12 mm GFs
1	6.72 × 10^−6^	7.28 × 10^−5^	−4.01 × 10^−6^	−1.94 × 10^−6^	−1.27 × 10^−5^	−3.41 × 10^−6^	2.57 × 10^−6^	−1.89 × 10^−7^
2	1.20 × 10^−6^	3.07 × 10^−8^	−6.03 × 10^−8^	1.15 × 10^−8^	2.26 × 10^−8^	1.60 × 10^−8^	1.06 × 10^−7^	1.99 × 10^−8^
3	−1.55 × 10^−5^	5.32 × 10^−8^	−9.92 × 10^−7^	−3.56 × 10^−9^	−3.00 × 10^−6^	−1.91 × 10^−7^	−1.09 × 10^−6^	3.16 × 10^−7^
4	−5.07 × 10^−6^	−8.89 × 10^−7^	−3.42 × 10^−7^	−1.08 × 10^−7^	9.23 × 10^−7^	6.73 × 10^−8^	−2.09 × 10^−7^	−7.39 × 10^−8^
5	2.67 × 10^−4^	−7.66 × 10^−3^	5.16 × 10^−4^	3.49 × 10^−4^	1.64 × 10^−3^	5.35 × 10^−4^	−8.57 × 10^−5^	1.68 × 10^−4^
6	−3.46 × 10^−4^	−1.86 × 10^−5^	3.11 × 10^−5^	−8.08 × 10^−6^	−3.85 × 10^−5^	−1.35 × 10^−5^	−2.08 × 10^−5^	−1.11 × 10^−5^
7	2.11 × 10^−3^	4.46 × 10^−4^	1.31 × 10^−4^	5.40 × 10^−5^	−2.81 × 10^−7^	−1.99 × 10^−5^	1.61 × 10^−4^	2.14 × 10^−5^
8	−8.18 × 10^−3^	2.19 × 10^−1^	−1.30 × 10^−2^	−1.20 × 10^−2^	−4.94 × 10^−2^	−1.54 × 10^−2^	−2.17 × 10^−2^	−2.58 × 10^−2^
9	1.35 × 10^−2^	7.06 × 10^−3^	−3.45 × 10^−3^	2.08 × 10^−3^	5.20 × 10^−3^	2.54 × 10^−3^	−4.83 × 10^−3^	1.30 × 10^−5^
10	35.03	34.60	2.72	2.77	2.25	2.10	1.59	1.57
$R^{2}$	0.78	0.95	0.86	0.78	0.65	0.48	0.92	0.95

**Table 6:** Comparison between the predicted values and measured values of *f_c_* and *ε_c_*.

Mix Number	6 mm GF Series				12 mm GF Series			
	Predicted Values of *f_c_* (MPa)	Change (%)	Predicted Values of *ε_c_* (×10^3^)	Change (%)	Predicted Values of *f_c_* (MPa)	Change (%)	Predicted Values of *ε_c_* (×10^3^)	Change (%)
S0G0	35.03	1.95	2.72	0.95	34.60	0.70	2.77	2.58
S0.4G0	35.08	−5.01	2.65	−0.82	36.46	−1.28	2.65	−0.82
S0.8G0	36.00	2.67	2.85	−0.69	35.53	1.34	2.78	−2.95
S1.2G0	38.33	0.64	2.97	0.54	37.85	−0.62	2.99	1.24
S0G0.2	34.40	0.47	2.62	0.66	35.42	−0.73	2.94	−4.20
S0.4G0.2	37.14	1.55	2.71	−3.90	38.49	1.42	2.98	5.57
S0.8G0.2	39.21	1.66	2.97	4.49	38.78	−2.58	3.25	−4.95
S1.2G0.2	41.18	−3.22	3.05	−1.12	42.34	1.80	3.60	4.09
S0G0.4	33.21	−4.47	2.74	−1.80	36.07	−0.79	2.99	−1.77
S0.4G0.4	36.85	5.98	2.87	3.67	39.10	1.59	3.02	2.03
S0.8G0.4	38.29	−4.39	3.07	−0.37	39.36	0.96	3.29	9.46
S1.2G0.4	38.10	3.34	3.00	−1.31	42.90	−1.60	3.63	−7.40
S0G0.6	35.98	2.05	2.87	0.26	37.49	0.83	3.26	3.60
S0.4G0.6	38.73	−2.02	2.92	1.05	39.22	−1.70	3.13	−5.88
S0.8G0.6	37.76	0.48	2.94	−3.17	38.20	0.48	3.23	−0.92
S1.2G0.6	33.62	−0.31	2.59	2.31	40.47	0.46	3.42	3.39

## Data Availability

Not applicable.
